# The cross talk of ubiquitination and chemotherapy tolerance in colorectal cancer

**DOI:** 10.1007/s00432-024-05659-9

**Published:** 2024-03-23

**Authors:** Ze Rong, Kaifeng Zheng, Jun Chen, Xiaofeng Jin

**Affiliations:** 1 Department of Chemoradiotherapy, the Affiliated People’s Hospital of Ningbo University, Ningbo, 315040 China; 2 Department of Biochemistry and Molecular Biology, Health Science Center, Ningbo, 315211 China

**Keywords:** CRC, Ubiquitination enzymes, Chemoresistance, Targeted therapy

## Abstract

Ubiquitination, a highly adaptable post-translational modification, plays a pivotal role in maintaining cellular protein homeostasis, encompassing cancer chemoresistance-associated proteins. Recent findings have indicated a potential correlation between perturbations in the ubiquitination process and the emergence of drug resistance in CRC cancer. Consequently, numerous studies have spurred the advancement of compounds specifically designed to target ubiquitinates, offering promising prospects for cancer therapy. In this review, we highlight the role of ubiquitination enzymes associated with chemoresistance to chemotherapy via the Wnt/β-catenin signaling pathway, epithelial–mesenchymal transition (EMT), and cell cycle perturbation. In addition, we summarize the application and role of small compounds that target ubiquitination enzymes for CRC treatment, along with the significance of targeting ubiquitination enzymes as potential cancer therapies.

## Introduction

Colorectal cancer (CRC) is the third most diagnosed cancer and the second leading cause of cancer-related deaths worldwide, accounting for over 1.9 million new cases and 935,000 new deaths worldwide (Sung et al. 2021). The heterogeneous and metastatic nature is the hallmark of CRC, with a poor prognosis (Van Cutsem et al. 2016). The prognosis of CRC is related to its stage at the time of diagnosis, with a 5-year survival rate of approximately 90% for stage I, 70% for stage II, 58% for stage III, and less than 15% for stage IV (Johnston 2005). Currently, adjuvant chemotherapy is the primary treatment for CRC patients, and systemic adjuvant chemotherapy based on fluoropyrimidine (5-fluorouracil [5-FU] or capecitabine) has been widely used in all patients with stage II and stage III CRC with high-risk clinicopathologic features (Messersmith 2017). A recent meta-analysis of 25 well-reported studies found that the 5-year relapse-free survival (RFS) estimates for patients with stage II and III CRC treated without adjuvant chemotherapy were 82.7% and 49.0%, respectively, compared to 79.3% and 63.6%, respectively, with adjuvant chemotherapy (Böckelman et al. 2015). However, the prognosis for these patients continues to be unfavorable, primarily due to the inherent resistance of these tumors to chemotherapy. Nonetheless, the overall survival of patients with colorectal cancer (CRC) has shown improvement in recent decades, thanks to the introduction of various chemotherapeutic agents despite their considerable drug resistance (Park et al. 2019). Consequently, it is necessary to summarize the potential mechanisms underlying the development of chemotherapy resistance during CRC treatment, which may have the potential to develop effective treatments to prevent chemotherapy resistance of CRC patients.

**Fig. 1:**
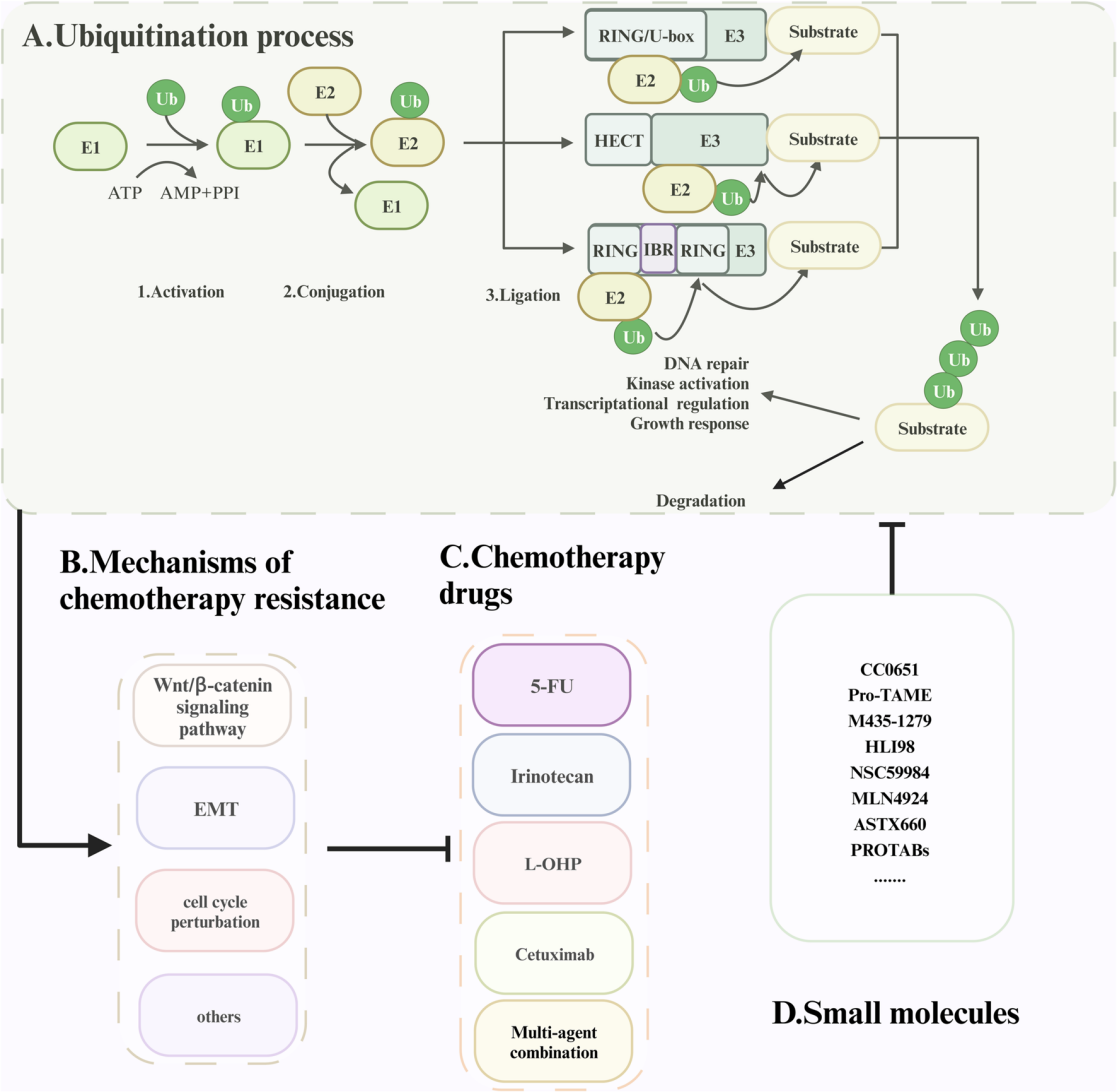
The flowchart illustrates the contents of the present review.** A** Ubiquitination process involves Ub, E1, E2, and E3 ubiquitin ligases. ATP activates the E1 enzyme and binds to Ub. The Ub-E1 intermediate then transfers the activated Ub to the E2 enzyme. Finally, Ub is transferred to a specific substrate by E3 ubiquitin ligases, which are categorized as the really interesting new gene (RING)-type E3 ligases, the U-box-type E3 ligases, the HECT-type E3 ligases, or the RBR-type E3 ligases. In RING and U-box E3 ubiquitin ligases, the Ub is transferred directly from the E2 enzyme to the substrate. HECT E3 ligase transfers Ub to the C lobe of HECT, and then Ub is transferred back to the substrate. RBR E3 ubiquitin ligase transfers Ub through the two RING domains. Subsequently, the substrate with the Ub linkage is destined for degradation or to participate in DNA repair, kinase activation, transcriptional regulation, and growth reactions. **B**, **C** ubiquitination enzymes are involved in the process of resistance to first-line chemotherapeutic agents, such as 5-FU, CPT-11, L-OHP, and Cetuximab, mainly through their involvement in the Wnt/β-catenin signaling pathway, EMT, cell cycle perturbation, and others. **D** Various small-molecule drugs that target ubiquitination enzymes are being developed for use in the clinical management of CRC

In eukaryotic cells, the ubiquitin proteasome system (UPS) is responsible for protein level control or activity regulation of many proteins in a highly selective manner (Chen and Dou 2010). Numerous studies have demonstrated the vital role of the UPS in CRC resistance to chemotherapy (Wang et al. 2022). Ubiquitin (Ub) is a β-grasp-fold protein with a compact overall structure and tight hydrogen bonds. It contains seven lysine residues involved in the formation of different types of Ub chains that have different roles in regulating and maintaining cellular homeostasis of the cell (Husnjak and Dikic 2012). Ubiquitination is a multifaceted enzymatic process that encompasses a series of regulated steps, involving the activation of ubiquitin through the ubiquitin-activating enzyme E1, the conjugation of ubiquitin through the ubiquitin-conjugating enzyme E2, and the subsequent transfer of the ubiquitin moiety to the target molecule facilitated by the ubiquitin protein ligase E3 (Han et al. 2022) (Fig. 1A). Notably, E3 ligases, as the core component of the UPS, can recognize the most specific substrates and are widely studied as potential targets to intervene in chemotherapy resistance, mainly through the Wnt/β-catenin signaling pathway, EMT, and cell cycle perturbation (Fig. 1B, C).

With the advances in the development of small molecules targeting various ubiquitination enzymes, it may be a promising treatment alone or in combination with other drugs to overcome the severe chemotherapy resistance in CRC (Fig. 1D). This review aims to elucidate the role of ubiquitination enzymes in the development of therapeutic resistance in colorectal cancer (CRC). Additionally, it provides a comprehensive analysis of the molecules examined in clinical trials for CRC treatment, with the intention of offering novel perspectives on strategies to overcome drug resistance.

## Types of ubiquitinated enzymes

### E1 ubiquitin-activating enzymes and E2 ubiquitin-conjugating enzymes

E1 enzymes (E1s), the first key enzymes in the ubiquitination process, can be shared in the process of ubiquitination, and there are only two kinds of E1 in humans (Varshavsky 2012). The adenosine 5'-triphosphate (ATP)-binding site is currently the sole identified means of inhibiting E1s. E1 triggers the activation of Ub through an ATP-dependent reaction, which necessitates the consumption of energy, resulting in the formation of a thioester-linked E1–Ub conjugate. Subsequently, the activated Ub is transferred to an E2 enzyme (E2s) via a trans-thiolation process involving an E2 Cys residue. E2s consist of 40 members and act as bridges to help the shuttle of Ub from E1s enzymes to E3 enzymes (E3s) or substrates during the ubiquitination process (Zhou et al. 2021a, b). To date, all E2s contain a conserved catalytic core domain of approximately 150 amino acids, termed the Ub-conjugating domain or UBC (Stewart et al. 2016). E2s can be divided into four classes based on having extra N- and/or C-terminal domains with E2-specific functions and enable specific interactions with particular E3s: class I E2s consist of a UBC domain, class II has an additional N-terminal domain, class III E2s have additional C-terminal domains, whereas class IV E2s contain both N- and C-terminal domains (Van Wijk and Timmers 2010). Some E2s have been shown to regulate the Wnt/β-catenin signaling pathway and drug transporter proteins to make CRC cells resistant to chemotherapeutic drugs (Huang et al. 2020).

### E3 ubiquitin ligases

E3s display specific recognition of substrates, which plays a critical role in UPS (Toma-Fukai and Shimizu 2021). More than 600 E3s have been identified in humans and classified into four categories based on their distinctive catalytic structural domains and Ub transfer characteristics: really interesting new gene (RING)-type E3 ligases, UFD2 homology (U-box)-type E3 ligases, and homologous to E6AP carboxyl terminus (HECT)-type E3 ligases or the RING-between-RING (RBR)-type E3 ligases(Buetow and Huang 20166).

The most abundant E3 ubiquitin ligases in humans are RING-type E3 ligases, named after its RING domain, which requires the chelation of two zinc ions (Zn^2+^) for its activity (Zhao et al. 2022; Morreale and Walden 2016). Remarkably, RING-type E3 enzymes can mediate the direct transfer of Ub from the E2 enzyme to the substrate, functioning as monomers, homodimers, heterodimers, or multiple subunits (Morreale and Walden 2016; Berndsen and Wolberger 2014). Among them, the anaphase-promoting complex/cyclosome (APC/C) and cullin–RING E3 ubiquitin ligase (CRL) are both composed of multiple subunits. All CRLs share a common feature with at least four common subunits, including the E2-binding RING protein, scaffold containing the cullin protein, substrate-recognized receptor, and articulation protein between the receptor and scaffold (Harper and Schulman 2021). The well-studied cullin protein family consists of CUL1 to CUL7, which bind to BTB proteins to specifically recognize substrates (Fouad et al. 2019). Moreover, multiple tripartite motif (TRIM) family proteins, which belong to a large class of molecules in the RBCC protein family that contain the RING structural domain, have been shown to play a critical role in chemotherapy resistance in CRC (Liang et al. 2019). U-box ligases, an E3 ubiquitin ligase domain structurally similar to the RING-type E3 ligase domain, link Ub to substrates in a single reaction, but their activity is not dependent on Zn^2+^ (Morreale and Walden 2016).

As for HECT-type E3s, consisting of approximately 30 have been identified in humans (Rotin and Kumar 2009). The HECT-type E3 ligase possesses a conserved catalytic HECT structural domain with an approximate molecular weight of 40 kDa, which is responsible for specific substrate recognition. Typically, the HECT E3 ligase facilitates substrate ubiquitination through a two-step process. Initially, the HECT domain interacts with the E2 enzyme and transfers Ub to the C lobe via a trans-thioesterification reaction. Subsequently, Ub is subsequently transferred to the substrate (Weber et al. 2019). Based on the N lobe structure, HECT E3s can be further divided into three subfamilies: the NEDD4 subfamily containing multiple tryptophan–tryptophan (WW) motifs, the HECT and RCC1-like structural domain (HERC) subfamily, and HECT E3s that retain different structural domains. Furthermore, HECT-type E3s have also been found to be associated with chemotherapy resistance. Both HECT-type E3 and RBR-type E3 require a two-step reaction to link Ub to the substrate. As a newly identified E3 ligase isoform, more than 10 RBR-type E3s have been identified in humans (Cotton and Lechtenberg 2020). In RBR E3 ligases, there are usually three structural domains: the RING1 domain, which is responsible for binding to Ub-containing E2; the RING2 domain, which transfers Ub to the substrate; and the zinc-binding intermediate-ring domain (IBR) (Qi et al. 2015).

Ubiquitination has been shown to play a pivotal role in the occurrence and progression of many cancers, as well as the advances showing that ubiquitination enzymes are associated with drug resistance in cancer.

## Mechanisms of chemotherapy resistance in CRC

Resistance of CRC to chemotherapy involves multiple mechanisms. In the following section, we describe in detail the mechanisms involved in ubiquitination enzymes in the context of chemotherapeutic drug resistance.

### Wnt/β-catenin signaling pathway

The Wnt/β-catenin signaling pathway plays a pivotal role in the initiation and progression of CRC. Wnt molecules interact with frizzled (FZD) receptors, belonging to a specific family, to modulate the activity of β-catenin, a crucial downstream protein that exerts significant influence on cellular processes (Shay et al. 2016). When Wnt ligands do not bind to the transmembrane FZD receptors or low-density lipoprotein receptor-related protein 5/6(LRP5/6), the Wnt/β-catenin signaling is in an ‘off’ state (Sawa et al. 2016). The β-catenin could be targeted for ubiquitination and degradation through the adenomatous polyposis coli complex, consisting of glycogen synthase kinase 3β (GSK3β), framework protein Axin, APC and casein kinase 1α (CK1α) (Drew et al. 2016). The APC complex-mediated ubiquitination of β-catenin inhibits its nuclear translocation and activation of its target genes, such as *c-Myc* (Bernkopf et al. 2019). However, when the Wnt ligand binds to the receptor in normal mature cells, CK1 and GSK3β are recruited to LRP5/6, resulting in the inability of the APC complex to degrade β-catenin and thus promoting target gene transcription (Dominguez-Brauer et al. 2016; Zarkou et al. 2018).

**Fig. 2:**
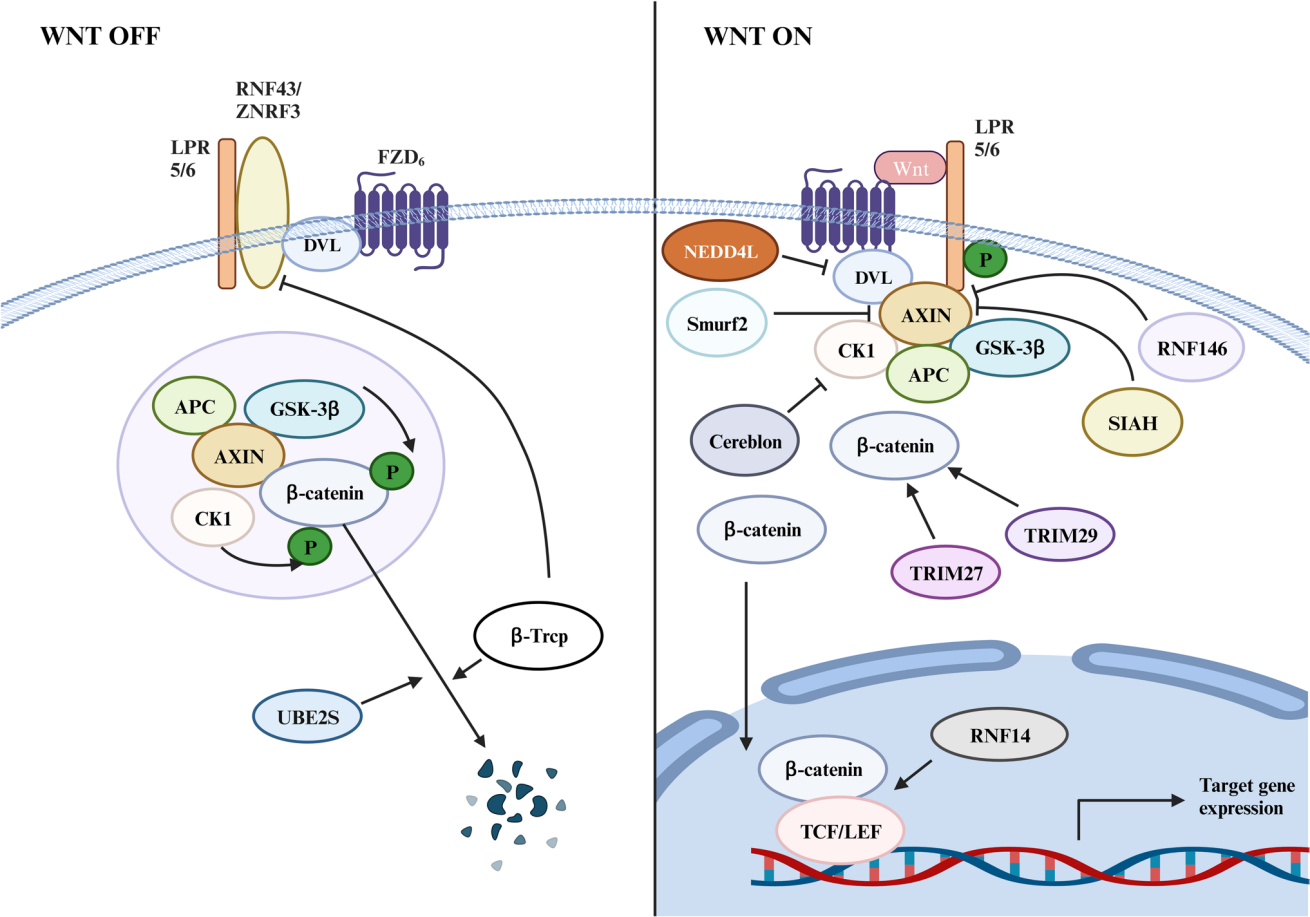
The role of ubiquitination enzymes in the Wnt/β-catenin signaling pathway of CRC. Under normal cellular conditions (in the absence of WNT), β-catenin in the cytoplasm undergo UPS-mediated degradation by interacting with a destructive complex (DC) consisting of APC, Axin, CK1 and glycogen synthase kinase 3 (GSK3-β). DVL removes FZD_6_ from the cell membrane via RNF43/ZNRF3 binding. LPR is also deleted. The ubiquitination enzymes β-TrCP and UBES2S promote the degradation of β-catenin. On the other hand, Wnt binding to the LPR and FZD_6_ prevents β-catenin degradation, the C-terminus of LRP is phosphorylated and the binding of Axin inhibits the interaction of β-catenin with DCs, leading to stable transfer of β-catenin to the nucleus that binds to the TCF/LEF transcription factors to activate transcription of Wnt target genes. Expression of β-catenin is promoted by TRIM29 and TRIM27. RNF14 promotes β-catenin binding to TCF/LEF. β-Catenin degradation has been shown to be inhibited by NEDD4L, Smurf2, CRBN, SIAH and RNF146

Notably, the Wnt/β-catenin signaling pathway has been reported to be involved in chemotherapeutic resistance of CRC by influencing the presence of highly metastatic cancer stem cells, regulating non-coding RNAs, and modulating the tumor microenvironment (Zhu et al. 2021a, b) (Fig. 2). Considering the regulatory role of ubiquitination on multiple elements of the Wnt/β-catenin signaling axis, it becomes crucial to provide a comprehensive overview of additional E3 ligases. Notably, ring finger 43 (RNF43) and its homolog, zinc and ring finger 3 (ZNRF3), are highly correlated ring finger proteins (RNFs) that function as negative modulators of Wnt/β-catenin signaling. Their mechanism involves facilitating the degradation of Wnt coreceptors FZD and LRP6, thereby impeding intracellular Wnt cascades and promoting tumor suppression (Jiang et al. 2015). However, it is interesting to note that β-TRCP positively regulated Wnt/β-catenin signaling by targeting ZNRF3 (Ci et al. 2018). As mentioned above, axin negatively regulates Wnt/β-catenin signaling by regulating the level of β-catenin, which is a key effector molecule. Knockdown of Smad ubiquitination regulatory factor 2(*Smurf2*) results in reduced activity of the β-catenin/Tcf reporter gene, specifically ubiquitinylated Lys (505) of Axin, inducing its degradation (Kim and Jho 2010). Moreover, the RING domain E3 ligase SIAH proteins promote the ubiquitination and proteasomal degradation of Axin by interacting with a VxP motif in Axin (Ji et al. 2017).

E3s RNF146 also mediates ubiquitination and degradation of Axin (Callow et al. 2011). Cereblon (CRBN), the substrate receptor of CRL4CRBN E3 ubiquitin ligase, induces ubiquitination and degradation of CK1-α, leading to an increase in Wnt/β-catenin signaling (Shen et al. 2021). Moreover, the ubiquitination of K6, K27, and K29 in Disheveled (Dvl2) by a neural precursor cell expressed, developmentally down-regulated 4-like (NEDD4L) could regulate β-catenin level (Ding et al. 2013b, a). In addition, β-catenin can be phosphorylated by GSK3β and then recognized by the Skp1–Cdc53/cullin–F-box-protein complex (SCF)/β-TrCP in the absence of Wnt, finally contributing to ubiquitination and degradation of β-catenin (Liu et al. 2018a, b). In addition, the ubiquitin-conjugating enzyme E2S (UBE2S) targets the ubiquitination and degradation of β-catenin (Li et al. 2018). Interestingly, TRIM27, a RING-type E3 ubiquitin ligase identified by differential display (EDD), directly targets GSK-3β for degradation, resulting in the stabilization of β-catenin (Shen et al.^2021^). TRIM29, a RING-type E3 ubiquitin ligase, indirectly increases β-catenin expression by upregulating CD44 expression, thus inducing activation of the Wnt/β-catenin signaling pathway (Wang et al. 2015). In addition, RNF14 stabilizes its interaction with TCF/LEF by promoting β-catenin recruitment; however, the specific mechanism needs to be elucidated (Wu et al. 2013).

### EMT

The process of epithelial–mesenchymal transition (EMT) involves the temporary induction of a quasi-mesenchymal cellular phenotype in epithelial cells, leading to the gradual loss of their characteristic cobblestone appearance in monolayer cultures and acquisition of a spindle-shaped mesenchymal morphology. It is important to note that despite this transition, the mesenchymal cells still possess the capacity to revert back to their original epithelial state (Dongre and Weinberg 2019). Cells undergoing EMT usually exhibit decreased expression of epithelial genes, including occludin, E-cadherin, and ZO-1, and increased expression of mesenchymal genes, such as fibronectin, N-cadherin, and vimentin (Lamouille et al. 2014). Changes in the expression of EMT-associated genes may influence physiological processes, including cell morphology, loss of adhesion, and the acquisition of stem cell-like features (Du and Shim 2016). EMT is a highly dynamic and reversible process during chemotherapy resistance (Vander Heiden and DeBerardinis 2017).

Numerous RING-type E3 ligases, particularly those within the TRIM family, have been implicated in the modulation of epithelial-mesenchymal transition (EMT). For example, TRIM21, an E3 ubiquitin ligase belonging to the TRIM family, exhibits a significant decrease in colitis-associated cancers and exerts a negative regulatory influence on colon carcinogenesis through the modulation of genes associated with tumor cell adhesion, such as E-calmodulin (Zhou et al. 2021a, b). Additionally, TRIM58 actively participates in EMT by regulating the expression of key transcription factors Snail and Slug, as well as cytoskeletal proteins vimentin and E-cadherin (Liu et al. 2018a, b). Moreover, *TRIM66* knockdown inhibited EMT by increasing E-cadherin expression and decreasing N-cadherin and waveform protein expression (He et al. 2019). TRIM27 has also been shown to promote EMT in CRC cells (Zhang et al. 2018). Another RING-type E3 ubiquitin ligase, TRIM37, enhances CRC metastasis by inducing EMT process (Hu and Gan 2017). S-phase kinase-associated protein 2 (Skp2) is a key component of the SCF complex belonging to the RING finger type and enhances cellular migration through ubiquitination and destruction of E-cadherin (Wang et al. 2012). TNF receptor-associated factor 6 (TRAF6) promotes the formation of the LC3B–ATG7 complex by interacting with LC3B and catalyzing K63-linked polyubiquitination, which is essential for the subsequent recognition of catenin beta1 (CTNNB1) for selective autophagic degradation to efficiently inhibit EMT efficiently (Wu et al. 2019). Moreover, the E3-ubiquitin ligase F-Box and WD repeat domain containing 7 (FBXW7) directly bind and degrade the EMT-inducing transcription factor zinc finger E-box-binding homeobox 2(ZEB2) in a phosphorylation-dependent manner (Li et al. 2019). Furthermore, Fbox45, TRIM62, retinoblastoma-binding protein 6 (RBBP6), and Hakai have been implicated in EMT (Xu et al. 2015; Chen et al. 2013; Xiao et al. 2019; Díaz-Díaz et al. 2017).

The HECT E3 ligase HERC3 has been found to facilitate the degradation of eukaryotic translation initiation factor 5A2 (EIF5A2) through K27- and K48-linked ubiquitination, utilizing its HECT domain. This degradation process has been shown to regulate the epithelial–mesenchymal transition (EMT) through the EIF5A2/TGF-/Smad2/3 signaling pathway (Zhang et al. 2022). Additionally, another HECT ligase, Itch, has been observed to positively regulate EMT by promoting TGF-β signaling via ubiquitination of Smad7 (Park et al. 2015). Furthermore, the WW domain-containing E3 ubiquitin protein ligase 2 (WWP2) has been identified as targeting Smad2, Smad3, and Smad7 for degradation, thereby exerting control over transforming growth factor β (TGFβ)-dependent transcription and EMT (Soond and Chantry 2011). Furthermore, the involvement of SNAIL in cancer progression is of paramount importance as it facilitates the process of epithelial–mesenchymal transition (EMT), while the degradation of SNAIL through ubiquitination by the HECT structural domain E3 ubiquitin ligase 1 (HECTD1) is responsible for mediating this phenomenon (Wang et al. 2020a, b). Additionally, β-TrCP1 has been demonstrated to participate in the ubiquitination of SNAIL (Zhong et al. 2013). Nevertheless, the precise mechanisms underlying drug resistance in EMT are still not fully understood, necessitating further comprehensive investigations in subsequent research.

### Cell cycle perturbation

The regulation of the cell cycle encompasses intricate mechanisms, such as the control of various cyclins, cyclin-dependent kinases, cell cycle checkpoints, and cell cycle signaling pathways. Historically, the eukaryotic cell cycle has been categorized into four distinct phases: G1, S, G2, and mitosis (M phase). During the G1 phase, cells undergo rapid synthesis of RNA and proteins, while also making preparations for DNA synthesis in the subsequent S phase. The S phase holds significant importance in the cell cycle as it involves the replication of DNA. The G2 phase denotes the stage where DNA replication has been completed, occurring prior to the initiation of mitosis. The M phase encompasses prophase, metaphase, anaphase and telophase, which collectively facilitate the precise and equitable division of chromosomes into two daughter cells (Sun et al. 2021). However, most cells do not enter the next cycle, but temporarily exist in a non-dividing state called the G0 phase.

**Fig. 3:**
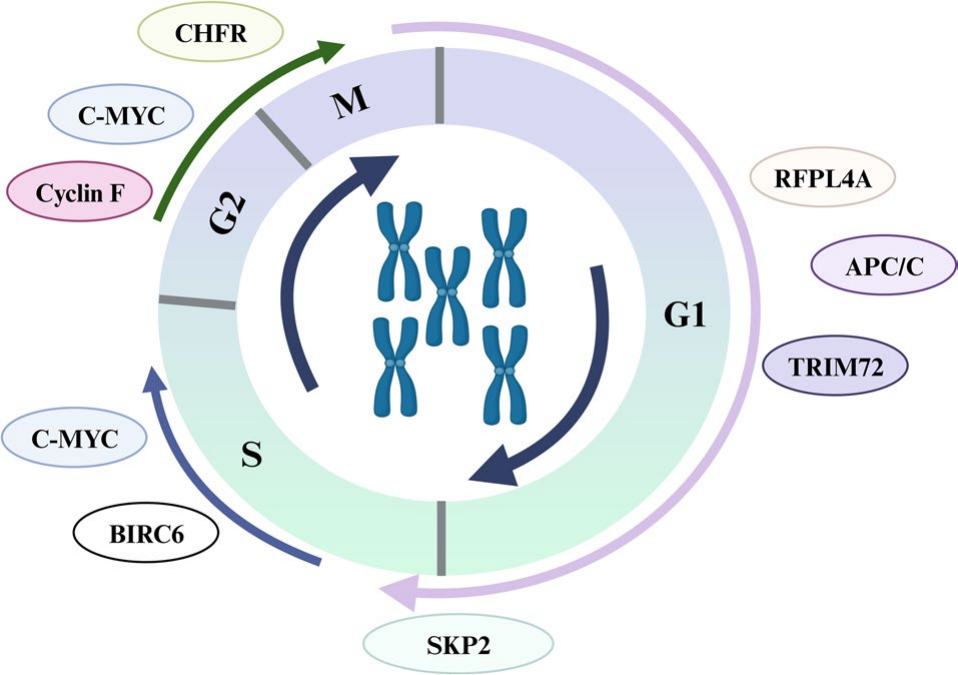
Cell cycle regulation of ubiquitination enzymes in CRC. The stages of the cell cycle are divided into four major phases: (1) G1 phase. (2) S phase. (3) G2 phase. (4) M phase. CHFR promotes G2/ -stage cell cycle arrest. c-Myc also promotes cell cycle arrest in G2/M phase and S phase. APC/C^CDC20^ regulates cell cycle progression through the M and S phase. Furthermore, SKP2 promotes cell cycle arrest at the G1/S transition. TRIM72 promotes G1-phase cell cycle arrest. Cyclin F inhibits the activity of transcription motors in the G2 cell cycle. RFPL4A has been shown to induce G1 arrest in CRC cells

Emerging evidence has shown that perturbation of cell cycle control enables continuous cell division primarily by impairing the ability of cells to exit the cell cycle, thus enhancing drug resistance (Matthews et al. 2022). Several RING-type E3 ligases have been shown to be involved in the regulation of this process (Fig. 3). The FHA domain of the RING-type E3 ligase checkpoint protein with forkhead associated and ring finger domains (CHFR) excludes cyclin B1 from the nucleus, leading to cell cycle arrest at G2/M, indicating a functional link between the anti-proliferative effects and checkpoint function of this tumor suppressor protein (Fukuda et al. 2008).

In contrast, knockdown of baculoviral inhibitor of apoptosis (IAP) repeat containing 6(BIRC6) inhibited cell proliferation, stalled the cell cycle in the S phase and downregulated the levels of cell cycle proteins A2, B1, D1 and E1, thereby sensitizing CRC cells to chemotherapy (Hu et al. 2015). Notably, the downregulation of c-Myc expression facilitated the inhibition of the cell cycle in CRC when exposed to cytotoxic drugs, leading to growth arrest in the G2/M and S phases (Abaza et al. 2008). APC/C is a multi-subunit CRL that regulates cell cycle progression through the M and S phases. Among these, activation of APC/C^CDC20^ subsequently mediates proteasomal degradation of cyclin B1 and securin, promoting chromosome segregation and late-phase onset (Singleton and Uhlmann 2017; Zhou et al. 2019). In addition, APC/C^CDH1^ mediates the ubiquitination and proteasomal degradation of a large number of mitotic and G1 regulators, including cyclin B1, PLK1, CDC20, FOXM1, and SKP2, facilitating irreversible mitotic exit and G1 maintenance (Li and Zhang 2009). The SCF complex, which belongs to the CRL family, is active throughout the cell cycle. Four substrates, SKP2, cyclin F, FBXW7 and β-TrCP, have been well characterized in cell cycle regulation (Ang and Wade Harper 2005).

Phosphorylated p27 is ubiquitinated by activated SCF–Skp2 ubiquitin E3 ligase in the late G1 phase, triggering its proteasomal degradation, which contributes to RB1 full phosphorylation and the G1/S transition (Roilo et al. 2018). In addition, abnormally high TRIM72 expression catalyzes K48-linked ubiquitination and degradation of cell cycle protein D1, leading to cell cycle arrest in the G1 phase (Fang et al. 2023). As cells enter G2 phase, cyclin F ubiquitinates and restricts the activity of E2F, the major and most critical transcription engine of the cell cycle (Clijsters et al. 2019). Furthermore, βTrCP has demonstrated its ability to modulate the stability of cyclin F, thereby facilitating the degradation of ribonucleotide reductase M2 (RRM2) and CP110, thereby exerting control over genome integrity and centrosome homeostasis. Additionally, βTrCP influences CDH1 activity, thereby impacting the cell cycle (D'Angiolella et al. 2012, 2010). FBXW7 regulates the cell cycle by inhibiting the destruction of c-Myc and cell cycle protein E (Dang et al. 2021; Mavrommati et al. 2018). In addition, abnormally high expression of RET finger-like protein 4A (RFPL4A), a RING-type E3 ligase, induces G1-phase retention in CRC cells, decreasing their sensitivity to chemotherapy; however, the specific mechanism remains to be elucidated (Naito et al. 2015). Moreover, X-linked inhibitor of apoptosis protein (XIAP), which has E3 ubiquitin ligase activity, upregulates the phosphorylation of PP2A to inhibit phosphatase activity, thus leading to the phosphorylation and activation of its downstream target c-Jun, which in turn contributes to enhanced cyclin D1 expression and cell cycle transition (Cao et al. 2013).

### Others

Multidrug resistance and membrane drug transporter proteins also influence drug resistance in CRC. P-glycoprotein (P-gp)/ABCB1 is an ABC transporter protein that acts as a vital determinant of the multidrug resistance phenotype of cancer cells (Srikant and Gaudet 2019). Knockdown of the RING-type E3 ligase SCF^Fbx15^ decreased the degradation and ubiquitination of P-gp, leading to resistance to chemotherapy (Katayama et al. 2013). Disturbance of apoptosis also contributes to resistance to chemotherapy in CRC. The E3 ubiquitin ligase TRIM25, which belongs to the TRIM family, inhibits CRC cell death by destabilizing caspase-2 and caspase-7, thereby mediating its resistance to chemotherapy (Nasrullah et al. 2023). Moreover, SCF-box and WD repeat domain-containing protein 7 (FBW7) regulate cellular apoptosis by mediating ubiquitination and degradation of myeloid cell leukemia sequence 1(MCL1) (Inuzuka et al. 2011). Mechanistically, TRAF6 ubiquitinates the K63 site of p53 to limit its mitochondrial translocation of p53, contributing to tumor development and drug resistance (Zhang et al. 2016). Homologous to the E6-associated protein carboxyl terminus domain containing 3 (HECTD3) promoted the polyubiquitination of solute carrier family 7 member 11 (SLC7A11) to trigger the degradation of SLC7A11, thereby promoting ferroptosis of CRC (Huang et al. 2023a, b). Interestingly, all the E3s described above are RING-type E3s; however, additional studies are needed to further elucidate other E3s in the chemotherapeutic mechanism of colon cancer (CC). An improved understanding of the mechanisms involved in the ubiquitination-associated resistance to chemotherapy in CRC may reveal novel therapeutic strategies.

## Chemotherapy drugs

It is widely acknowledged that CRC exhibits diverse responses to chemotherapy agents, including 5-FU, irinotecan (CPT-11), oxaliplatin (L-OHP), and cetuximab, with drug resistance being a prevalent occurrence. Consequently, this section aims to elucidate the interplay between drug resistance and ubiquitination in CRC.

### 5-FU

5-FU, an essential component of palliative and adjuvant systemic chemotherapy for CRC, is a synthetic fluorinated pyrimidine analog that replaces hydrogen with fluorine at the C-5 position of uracil (Vodenkova et al. 2020). 5-FU was one of the first chemotherapeutic agents reported to have anticancer activity. In CRC, treatment with 5-FU or other fluoropyrimidines (FPs) has been the backbone of systemic therapy since 1990 (Morawska et al. 2018). 5-FU is absorbed into the cell and activated to 5-FU deoxyribonucleotide for subsequent reactions; it exerts its antitumor effect by inhibiting thymidylate synthase (TS), preventing the conversion of deoxyuridine (dUMP) methylation to deoxythymidine (dTMP), and disrupting DNA replication (Siddiqui et al. 2019). It can also replace more than 50% of the uracil incorporated into RNA, inhibiting RNA synthesis (Shelton et al. 2016). Unfortunately, the use of oral 5-FU alone was abandoned because of its unpredictable gastrointestinal absorption and significant changes in pharmacokinetics (Saif et al. 2009). To maximize anticancer effects and minimize toxic effects, 5-FU is often used in combination with other drugs, such as folinic acid (Leucovorin, LV), bevacizumab, etc. (Thirion et al. 2004). Although new cancer therapies, such as targeted drug therapy, have made significant progress recently, 5-FU remains one of the most influential and commonly used drugs in CRC treatment and is a significant component of combination chemotherapy regimens (Sargent et al. 2009).

E3 ubiquitin ligases have been shown to play a role in the efficacy of 5-FU in patients with CRC. A few of these promote the therapeutic effects of 5-FU. For example, membrane-associated RING-CH-1 (MARCH 1), a RING-type E3 ubiquitin ligase whose expression is targeted by 5-FU, and the consequent downregulation of the PI3K/AKT pathway impact the progression of EMT (Wang et al. 2021). However, most E3 ubiquitin ligases directly contribute to 5-FU resistance. Inhibitors of apoptosis (IAPs), which belong to the RING-type E3 ubiquitin ligase family, have been shown to mediate 5-FU resistance (Oberoi-Khanuja and Rajalingam 2012). The expression level of XIAP increased with the number of 5-FU treatments, ultimately promoting the development of drug resistance (Flanagan et al. 2015). Inhibition of XIAP expression enhances the sensitization of CRC cells to 5-FU (Zhao et al. 2017). High expression of cIAP1 and cIAP2 was significantly associated with poor prognosis in patients with CRC treated with 5-FU, and further studies revealed that downregulation of cIAP2 effectively enhanced 5-FU sensitivity through the apoptotic pathway (Crawford et al. 2021). Ubiquitination and degradation of SMAD Family Member 4(SMAD4) increased upon physical interaction with TRIM47, leading to upregulation of the C–C motif chemokine ligand 15(CCL15) expression and chemotherapy resistance in response to 5-FU therapy (Liang et al.^2019^). The DNA damage-activated RING-type E3 ubiquitin ligase RAD18 was elevated after 5-FU treatment. Inhibition of its expression significantly attenuates proliferation and promotes apoptosis, thereby enhancing cellular radiosensitivity and 5-FU sensitivity. In contrast, elevated expression levels can induce DNA damage repair and promote drug resistance formation (Yan et al. 2019). UBE2M plays a role in resistance to 5-FU by regulating the expression of β-catenin (Xu et al. 2020).

Capecitabine is an oral fluoropyrimidine carbamate produced by the modified administration of 5-FU. It inhibits DNA synthesis through a series of enzyme-catalyzed reactions that convert it to 5-FU (Mehta et al. 2018). A large clinical trial demonstrated that capecitabine shows at least comparable efficacy and a good safety profile to push 5-FU in the treatment of metastatic CRC (Yamaguchi et al. 2006). Cancer cells with high nuclear factor erythroid 2-related factor 2 (NRF2) expression levels are less sensitive to chemotherapeutic agents, and multiple RING-type E3 ubiquitin ligase complexes, KEAP1-CUL3-RBX1, β-TrCP-SKP1-CUL1-RBX1, and HRD1, mediate ubiquitination and proteasomal degradation of NRF2, affecting the relationship between NRF2 overexpression and increased 5-FU resistance in CRC (Homma et al. 2009). The HECT structural domain and RCC1-like structural domain 5 (HERC5), a HECT type E3 ubiquitin ligase, were shown to degrade C-terminal-binding protein 1 (CtBP1) by ubiquitination in CRC cells. In contrast, HERC5 expression is downregulated in CRC, promoting the accumulation of CtBP1 and the formation of transcriptional complexes that inhibit apoptotic signaling and promote tumorigenesis. Interestingly, when inhibitors of HERC5 were combined with capecitabine, the inhibitory effects on cell proliferation and tumor growth were much more robust than when using those drugs alone (Zhu et al. 2021b, a).

### CPT-11

CPT-11 is the first topoisomerase I inhibitor approved for cancer treatment (Martino et al. 2017). CPT-11,7-ethyl-10-[4-(1-piperidinyl)-1-piperidinyl]-carbonyloxycamptothecin is a pentacyclic alkaloid that undergoes structural changes depending on the physiological pH of the cellular environment (Tsilimigras et al. 2017). CPT-11 forms ternary CPT-11-topoisomerase I-incision DNA complexes in vivo in the form of collisions with replication forks that stall them, leading to double-strand break (DSB) formation and ultimately apoptosis (Stenvang et al. 2013). Interestingly, the dose dependence of CPT-11 increased with increasing cellular topoisomerase concentration, which directly affected the sensitivity of cells to CPT-11 (Burris and Fields 1994). CPT-11 is hydrolyzed in the body by carboxylesterases and converted in the liver to SN-38, which is 100–1000 times more toxic than CPT-11 (Rivory and Robert 1995). In contrast, SN-38 is converted to SN-38 glucosinolate (SN-38G) by UDP-glucuronosyltransferase UGT1A1 in an enzymatic reaction and is inactivated by β-glucuronidase hydrolysis after intestinal excretion (Morton et al. 1999). The correlation between the resistance of CRC tissues to CPT-11 monotherapy and ubiquitination remains relatively limited. Ubiquitin-conjugating enzyme 2C (UBE2C), which is highly expressed in most CRC patients, not only promotes the growth rate of CRC cells, but also enhances resistance to CPT-11 (Cacciola et al. 2016). It has been reported that the Cullin2/ElonginB-CIS complex,which belongs to the CRL, rendered cells more resistant to CPT-11 by promoting the degradation of the pro-apoptotic protein Bim (Ambrosini et al. 2009). Furthermore, upregulation of FBXW7 attenuated the response to CPT-11 by significantly reducing c-Myc expression (Izumi et al. 2017). In addition, downregulation of c-IAP1 and c-IAP2, which leads to the inhibition of NF-κB activation, could enhance the chemosensitivity of CRC cells to CPT-11 (Yu et al. 2014). Interestingly, CPT-11 regulates several ubiquitination enzymes. CPT-11 inhibits the expression of Bcl-x and XIAP and promotes p53 non-dependent apoptosis in CRC cells (Ravi et al. 2004). Moreover, CPT-11 inhibits mouse double minute 2(MDM2), thereby releasing p53 and blocking G2/M phase apoptosis (Lee et al. 2019).

### L-OHP

Oxalato(trans-(-)-1,2-cyclohexanediamine)platinum(II), a third-generation platinum drug, was introduced in 2000 for the treatment of CRC and has gradually been explored for its therapeutic effects in other cancers. L-OHP acts intracellularly after its intravenous administration. It usually interacts with DNA-based nucleophilic molecules, forming an intra-strand adduct between two adjacent guanine residues or between guanine and adenine, disrupting DNA replication and transcription (Vander Heiden and DeBerardinis 2017). Interestingly, L-OHP–DNA adducts are not recognized and repaired by the mismatch repair system (MMR), resulting in tumors with a mismatch repair system (MMR) deficiency being more sensitive to L-OHP than the first-generation platinum drug cisplatin(Ahmad 2010). Unfortunately, intrinsic or acquired resistance to L-OHP remains a major impediment to achieving therapeutic benefits in CRC patients. Exploration of resistance mechanisms to L-OHP has become a hot topic in recent years (Martinez-Balibrea et al. 2015). The discovery of the correlation between ubiquitination enzymes and L-OHP resistance has helped in the development of new therapies aimed at overcoming this resistance. RAD6 with E2 ubiquitin-coupled activity and its homolog RAD18 with E3 ubiquitin ligase activity are essential for platinum-based chemotherapy-induced trans-lesion synthesis or post-replication repair. Inhibition of both is a potential new strategy for the treatment of chemotherapy in L-OHP-resistant CRC cells (Sanders et al. 2017).

In TGFβ signaling, the ubiquitination function of Smurf2 regulates the expression of inhibitor of differentiation 1(ID1) in CRC, which in turn causes resistance to L-OHP (Niu et al. 2021). In the p53 signaling pathway, MDM2 interacts with cyclophilin B (CypB), thereby enhancing p53 ubiquitination and degradation. This mechanism is also thought to contribute to the poor prognosis of L-OHP resistance (Choi et al. 2018). Furthermore, c-Myc promotes the transcriptional regulation and expression of WD-repeat protein 43(WDR43), a mechanism that facilitates the binding of WDR43 to ribosomal protein L11 (RPL11) and enhances the ubiquitination of p53 by MDM2, which reduces the stability of p53 proteins and induces chemotherapy resistance in CRC cells (Di et al. 2023). Several studies have demonstrated that the upregulation of XIAP, which mediates apoptosis, in CRC is also responsible for L-OHP-acquired resistance. When XIAP is inhibited, cells regain sensitivity to L-OHP-mediated apoptosis (Hua et al. 2018). In addition, the lack of E3 ubiquitin ligase FBXW7 promotes acquired resistance to L-OHP in CRC cells (Li et al. 2015). Exosome-mediated circ-FBXW7 increases L-OHP-induced apoptosis and inhibits L-OHP efflux, providing a new strategy to address L-OHP resistance in patients (Xu et al. 2021). Interestingly, the target protein cryptochrome2 (CRY2) was recognized by FBXW7. Although CRY2 upregulation caused by FBXW7 downregulation may be a novel prognostic biomarker, *CRY2* knockdown increased the sensitivity of CRC to L-OHP (Fang et al. 2015). BRCA1-associated RING domain protein 1(BARD1), a RING-type E3 ubiquitin ligase, forms a positive mutually regulated ternary complex with breast cancer type 1 susceptibility protein (BRCA1) and mammalian metallothionein-2A (MT2A). High expression of MT2A greatly increases CRC cell resistance to L-OHP and increases CRC cell proliferation and viability (Zhao et al. 2020). In addition, TRAF6, a RING-type E3 ubiquitin ligase that binds and promotes enhancer of zeste homolog 2(EZH2) degradation, is inhibited by TRIM25, thereby promoting the stabilization of EZH2 and the characteristic state of CRC stem cells, promoting the resistance of cancer tissues to L-OHP (Zhou et al.2021a, b). Moreover, TRIM25 is involved in the negative regulation of caspase-2 in different CRC cell lines, a phenomenon that protects tumor cells from chemotherapeutic drug-induced apoptosis, and may serve as a novel mechanism of drug resistance in CRC (Nasrullah et al. 2019).

In addition, cisplatin, one of the previous generations of platinum-based drugs, is still used clinically for the treatment of CC by producing DNA lesions, blocking proliferative function, and promoting apoptotic cell death. Increased E3 ubiquitin ligase XIAP induces cisplatin resistance in CRC by activating the PI3K/Akt pathway, which is reversed when XIAP is inhibited (Xiong et al. 2017). Downregulation of the nucleotide excision repair (NER) member ubiquitin ligase cullin (CUL) 4A, which belongs to the RING-type E3 family, effectively improves cisplatin sensitivity but mediates trabectedin resistance (Englinger et al. 2017). TRIM8 overexpression inhibits p53 stability and activity, leading to cisplatin resistance (Mastropasqua et al. 2017). Furthermore, upregulation of Mcl-1 reduces cisplatin sensitivity in *HUWE1* knockout mice (Myant et al. 2017).

### Cetuximab

Cetuximab is a chimeric human murine derivative IgG1 monoclonal antibody (mAb) directed against the ligand-binding structural domain of the bound epidermal growth factor receptor (Kim 2004). The affinity of cetuximab to the epidermal growth factor receptor (EGFR) is much greater than that of any of the endogenous ligands, which, in turn, inhibits the activation of the receptor tyrosine kinase and associated downstream signaling, thereby exerting an antitumor effect (Humblet 2004). Cetuximab is currently approved for use in combination with chemotherapy in patients with CRC, non-small cell lung cancer (NSCLC), and head and neck cancer. The therapeutic efficacy of cetuximab is often compromised by acquired drug resistance; however, the mechanisms underlying this phenomenon are still unknown. Computer simulations have shown that multiple ubiquitination-related protein gene polymorphisms are involved in EGFR turnover and predict the efficacy of cetuximab, such as the ubiquitin-binding enzyme UBE2M and ubiquitin-conjugating enzyme E2L3 (UBE2L3), which may affect secondary resistance to cetuximab in metastatic CRC (Stintzing et al. 2015). Moreover, previous reports have shown that resistance to cetuximab in CRC is associated with increased expression of c-Myc, which significantly reduces apoptosis (Boos et al. 2022). Interestingly, inhibition of glutamine transporter protein solute carrier 1 family member 5 (SLC1A5) increased EGFR degradation via the ubiquitin–proteasome pathway, thereby significantly enhancing the inhibitory effect of cetuximab on CRC proliferation (Ma et al. 2018). Casitas B spectrum lymphoma-b (Cbl-b), a RING-type E3 ubiquitin ligase, regulates cetuximab sensitivity through the ubiquitin–proteasome system in human gastric cancer cells (Yu et al. 2016).

### Multi-agent combination

Indeed, multi-agent combination chemotherapy is the central tenet of all therapies in patients with CRC. The concomitant phenomenon is often the emergence of simultaneous resistance to multi-drug combinations, which poses a significant problem for treatment.

FOLFOX, a combination regimen of folinic acid (FnA; “FOL”), 5-FU, and L-OHP, is a standard care strategy for the development of stage II/III CRC and liver metastases (Allegra et al. 2013). FOLFOX, L-OHP, and 5-FU were able to produce additive or synergistic anticancer activity, with stronger anticancer effects than when used alone. Interestingly, FnA enhanced the sensitivity of cancer cells to 5-FU (Matt et al. 2011). For clinical treatment, FnA and OxP are first administered to patients simultaneously via intravenous infusion, followed by intravenous 5-FU to achieve optimal efficacy (Kuebler et al. 2007). Although FOLFOX treatment has improved survival rates for patients with CRC, it is associated with low efficacy, dose-limiting side effects, poor quality of life, and increased costs (André et al. 2004). In addition, drug resistance to FOLFOX is still an inevitable phenomenon (Chang et al. 2020). It is essential to elucidate the mechanisms of resistance to explore new combination therapies for multi-drug resistance in CRC. Multiple ubiquitination enzymes have been found to be involved in FOLFOX treatment resistance in CRC patients. The ubiquitin-conjugating enzyme E2M (UBE2M) mediates 5-FU and L-OHP resistance in CRC cells through the Wnt/β-catenin signaling pathway (Xu et al. 2020). Furthermore, a novel RING-type E3 ubiquitin-linked enzyme, RNF126, promoted CRC progression and induced FOLFOX treatment resistance by enhancing p53 ubiquitination and degradation (Wang et al. 2020a, b). Another RING-type E3 ligase, TRIM6, which is highly expressed in patients with CRC, was inhibited and subsequently induced cell cycle arrest in the G2/M phase and increased sensitivity to 5-FU and L-OHP (Zheng et al. 2020). Moreover, FBXW7 binds to and degrades the transcription factor ZEB2, thereby inhibiting the resistance of CRC cells to 5-FU and L-OHP chemotherapeutic agents (Li et al.^2019^). In contrast, FBXW7 inhibition by miR-92a-3p reduces mitochondrial apoptosis and induced resistance (Hu et al. 2019). In addition, CDK2-associated cullin structural domain 1 (CAC1), a member of the CRL family, acts as a cell cycle regulator whose expression positively correlates with P-gp and MRP-1 protein expression and promotes the development of 5-FU and L-OHP resistance (Chen et al. 2019). RANBP2 and C3HC4 containing zinc finger 1 (RBCK1), a RING-type E3 ubiquitin ligase belonging to the IAPs family, and inhibition of their expression levels increased the sensitivity of CRC cells to 5-FU and L-OHP. After performing protein back-complementation, drug resistance in CRC cells was improved (Liu et al. 2019).

## Targeting E3 ligases for CRC therapy

The modulation of crucial cellular pathways by ubiquitination enzymes makes them appealing targets for cancer treatment. The inhibition of these enzymes has shown promising effect on the field of cancer therapy (Lee et al. 2016). The research on drug discovery based on E3 ubiquitin ligase is progressing towards a hopeful future, with multiple biotech startups and companies developing small-molecule drugs targeting E3 ubiquitin ligase in various preclinical and clinical stages. The following section provides a description of small-molecule compounds or antibodies that specifically target ubiquitination enzymes, which are currently being investigated in various preclinical cancer models and ongoing clinical trials (Table 1).

**Table 1:** Small molecules targeting ubiquitination enzymes in CRC

Small compound	Targeted E3 ligase	Mode of action	Targeted cancer conditions	References
Auranofin	UBA1	Enhances UBA1 interactions with E2 ubiquitin-conjugating enzymes	rheumatoid arthritis	(Yan et al. 2023)
Arctigenin	UBC12	Inhibits UBC12 enzyme activity	NA	(Chen et al. 2023)
M435-1279	UBE2T	Blocks UBE2T-mediated degradation of RACK1 to inhibit the Wnt/β-collagen signaling pathway	Glioblastoma, gastric cancer	(Yu et al. 2021)
UM0130646	UBCH10	Inhibits UBCH10 activity and prevents p21 degradation	NA	(Pelletier et al. 2023)
RG7112	MDM2	Binds MDM2 and inhibits MDM2-p53 interaction	advanced solid tumors, leukemia, liposarcoma	(Tovar et al. 2013)
Nutlin	MDM2	Binds to the binding pocket of p53 in MDM2, and competitively inhibits MDM2–p53 interactions	Osteosarcoma xenograft, lymphomas	(Ray-Coquard et al. 2012; Andreeff et al. 2016; Psatha et al. 2023)
RG7388	MDM2	Binds MDM2 and inhibits MDM2–-p53 interaction	Solid tumors, AML, neuroblastoma, breast cancer	(Ding et al. 2013a, b) (Yu et al. 2014)
AMG‐232	MDM2	Binds MDM2 and inhibits MDM2–p53 interaction	AML, advanced solid tumors, glioblastoma metastatic melanoma, multiple myeloma	(Rew and Sun 2014; Gluck et al. 2020)
RITA	MDM2	Binds specifically to p53 and induces a conformational change that inhibits the interaction of MDM2 with the p53 protein	Fibrosarcoma and lymphoma cell lines and cervical carcinoma xenograft	(Issaeva et al. 2004; Zhao et al. 2010)
SAR405838	MDM2	High-affinity Binds MDM2 and inhibit the MDM2 interaction with the p53 protein	Malignant neoplasm, lymphoma advanced solid tumo rs	(de Jonge et al. 2017)
APG-115	MDM2	Binds MDM2 blockade of its inhibitory effect on p53	Advanced solid tumors, lymphoma, advanced liposarcoma, AML T-prolymphocytic Leukemia	(Rasco et al. 2019)
Milademetan (DS‐3032b)	MDM2	Disrupts MDM2–p53 interaction	Advanced solid tumors, lymphomas, AML, dedifferentiated liposarcoma	(Takahashi et al. 2021; Sekiguchi et al. 2023)
CGM097	MDM2	Binds MDM2 inhibiting MDM2–p53 interaction	Advanced solid tumors	(Bauer et al. 2021; Maser et al. 2020)
Siremadlin (HDM201)	MDM2	Binds MDM2 preventing MDM2–p53 interaction	Liposarcoma, AML, Advanced/metastatic soft tissue sarcoma, CRC	(Stein et al. 2022)
BI 907828	MDM2	Disrupts MDM2–p53 interaction	Advanced solid tumors, glioblastoma, pancreatic neoplasm dedifferentiated liposarcoma	(LoRusso et al. 2021)
MK‐8242	MDM2	Binds to MDM2 and prevent HDM2–p53 interaction	Solid tumors, AML	(Ravandi et al. 2016)
melatonin	MDM2	Inhibits phosphorylation of MDM2, enhances acetylation of p53	breast cancer	(Proietti et al. 2014)
Evodiamine	MDM2	Inhibits MDM2	Glioblastomas	(Sharma and Kumar 2023)
Sanguinarine	MDM2	Inhibits MDM2	Glioblastomas	(Sharma and Kumar 2023)
DR6-NLS-TAD10-B3	HDM2	Binds to HDM2 and prevent HDM2–p53 interaction	NA	(Mukherjee et al. 2023)
Compound A	SKP2	Prevents SKP2 association in SCF complex	Multiple myeloma cell lines	(Chen et al. 2008)
Compound #25 (C25)	SKP2	Prevents SKP2 interaction with adapter SKP1 and inhibit E3ligase activity of SKP2	Liver, lungs, prostate and osteosarcoma cell lines	(Chan et al. 2013)
DT204	SKP2	Prevents SKP2 interaction with Cullin1 and Commd1	Myeloma tumors (murine model	(Malek et al. 2017)
C series compound (C1, C2, C16, C20)	SKP2	Inhibits Cks1 activity to destabilize SKP2–p27 interaction	Melanoma cell lines	(Wu et al. 2012)
Curcumin	SKP2	Downregulate SKP2 expression	Prostate cancer, pancreatic cancer	(Dhillon et al. 2008; Mahammedi et al. 2016)
Dioscin	SKP2	Promotes SKP2–CDH1 interaction necessary for CDH1‐medaited degradation of SKP2	CRC cell lines	(Zhou et al. 2020)
Citrus grandis 'Tomentosa'	SKP2	Inhibits Skp2 activity, downregulates Skp2 protein levels and promotes p27 accumulation	NSCLC	(Huang et al. 2023b, a)
Oridonin	FBXW7	An agonist of Fbxw7, enhances the degradation of c‐MYC	Leukemia and lymphoma cell lines	(Huang et al. 2012)
MLN4924	CRLs	Inhibits NAE to prevent cullin ring neddylation	Advanced solid tumors, AML, MDS, lymphoma	(Adès et al. 2022; Sekeres et al. 2021; Swords et al. 2015)
NSC1892	CRL4^DCAF4^	Disrupts CUL4A/B‐Disrupts CUL4A/B-DDB1 interaction and prevents CRL4^DCAF4^ formation. Leading to the accumulation of the ST7, p21, and p27 proteins	CRC cell lines	(Yang et al. 2020)
Z1391232269	CRL4D^CAF1^	Targets and inhibits DCAF1	NA	(Li et al. 2023)
Pro‐Tame	APC/C	Binds APC/C and inhibits IR tail-dependent recruitment of CDC20 and CDH1	N/A	(Zeng et al. 2010)
Apcin	APC/CCDC20	Binds the D‐box-binding site of CDC20 and prevents recruitment of APC/CDC20 substrate	N/A	(Sackton et al. 2014)
GS143	β‐TrCP	Inhibits β‐TrCP ubiquitination of IKBα	N/A	(Nakajima et al. 2008)
C25‐140	TRAF6	Inhibits TRAF6‐Ubc13 interaction to reduce TRAF6 E3 ligase activity	A study conducted in Autoimmune condition	(Brenke et al. 2018)
Gliotoxin	LUBAC	Binds the RBR domain of HOIP and inhibits linear ubiquitin chain formation	N/A	(Sakamoto et al. 2015)
Compound	LUBAC	Binds the active cysteine of HOIP, and inhibits the formation of thioester bonds	NA	(Johansson et al. 2019)
HOIPINs	LUBAC	Binds active Cys885 and other associated residue of HOIP and inhibit linear ubiquitin chain formation	NA	(Oikawa et al. 2020)
SMAC mimetic: AT‐406 (DEBIO1143)	IAPs	Binds XIAP and cIAPs to induce the degradation of cIAP1 and the activation of caspases	Solid tumors, AML, lymphoma, squamous cell carcinoma	(Hurwitz et al. 2015; Sun et al. 2020; Cai et al. 2011)
SMAC mimetic: GDC‐0152 (Compound 1)	IAPs	Binds BIR3 domain of XIAP and the CIAP domain and the BIR domain of ML-IAP and induces the activation and degradation of cIAP by caspase 3/7	Solid cancers	(Flygare et al. 2012)
SMAC mimetic: LCL161	CIAPs	Binds the BIR3 domain of the cIAPs and induces autoubiquitination and degradation of these domains	Solid tumors, multiple myeloma, breast cancers, small cell lung cancer	(Infante et al. 2014; Bardia et al. 2018)
SMAC mimetic: Birinapant (TL327711)	CIAPs	Binds BIR3 domain of the cIAPs in order to induce their autoubiquitination and degradation	Solid tumors, MDS, ovarian cancers, Neck squamous cell carcinoma	(Benetatos et al. 2014; Amaravadi et al. 2015)
PRT4165	PRC1	Inhibits PRC1-induced ubiquitination of H2A and the H2AX gene	NA	(Amaravadi et al. 2015)
GW‐516	PRC1	Catalytically inhibits the RNF2 component of the PRC1 protein	Prostate cancer cell lines	(Su et al. 2019)
RB‐3	PRC1	Binds RING1B/BMI1 complex and induces conformational changes that perturb their interaction with nucleosomes	Leukemia cell lines	(Shukla et al. 2021)
conjugate 955	KEAP1	Recruits KEAP1 and degrades CDK9	NA	(Pei et al. 2023)
AZD7762	CRBN	Promotes CRBN binding to BAG3 and degradation of BAG3	NA	(Liao et al. 2023)
MyoMed-205	MuRF1	Inhibits MuRF1, mitigates FoxO1 activation, inhibits MuRF2, and increases phospho (ser473) Akt protein levels	disuse-induced diaphragmatic dysfunction	(Ribeiro et al. 2023)
inh-2	RNF5	Inhibits RNF5 activity and increases autophagy levels	cystic fibrosis	(Sondo et al. 2018)
compound 16	RNF5	Inhibits RNF5 activity and increases autophagy levels	cystic fibrosis	(Brusa et al. 2023)

### Small molecules that specifically target ubiquitination enzymes in CRC

Inhibitors targeting the Wnt/β-catenin signaling pathway remain promising antitumor therapeutic agents. M435-1279, a novel UBE2T inhibitor that inhibits the over-excitation of the Wnt/β-catenin signaling pathway by blocking the UBE2T-mediated degradation of RACK1, has been shown to play a role in gastric cancer and provides insights for the treatment of CC (Anastas and Moon 2013).

Specific targeting of MDM2 or mouse double minute X(MDMX) is one approach for the treatment of CRC through the p53 pathway (Wade et al. 2013). HLI98, a novel small molecule targeting the ubiquitin ligase MDM2, specifically inhibits MDM2 to activate the p53 signaling pathway to inhibit cancer occurrence and development in CRC (Vassilev 2007). Gu screened novel inhibitors that antagonize MDM2 protein-XIAP mRNA interactions and inhibit MDM2 stability while blocking XIAP translation levels, producing anti-proliferative and pro-apoptotic effects. It is interesting to note that the inhibitors degraded MDM2, leading not only to inhibition of XIAP expression, but also activated p53 and induced apoptosis in p53 wild-type cancers (Gu et al. 2016). It is worth noting that NSC59984, a small molecule that induces degradation of mutated p53 protein through the MDM2 and ubiquitin–proteasome pathways, has been used in combination with CPT-11 to synergistically induce cell death in CRC cells expressing p53 mutant, indicating a promising therapeutic strategy (Zhang et al. 2015). MMRi71, a novel small-molecule compound with dual targeting of MDM4/FTH1, not only accumulates p53 proteins in wt-p53 bearing cancer cells, but also effectively kills leukemia cells that are p53 deficient. Whose development provides a prototypical structure for potential anticancer therapeutics (Lama et al. 2022).

Good progress has been made in the development of small-molecule compounds to target key subunits of SCF or APC/C complexes in CRC (Milhollen et al. 2011). MLN4924, a novel inhibitor targeting the ubiquitin ligase SCF E3, sensitizes CRC cells to irradiation by inducing cell cycle arrest and increasing apoptosis and DNA damage, which can be used in combination therapy with chemotherapeutic agents on the basis of broken DNA levels in the future (Wan et al. 2016). In contrast, MLN4924 inactivates CRL and leads to the accumulation of CRL substrates, thereby inhibiting the growth of tumor cells in vitro and in vivo. Because of its favorable results, MLN4924 has entered several clinical trials for anticancer therapies (Tan et al. 2011). It is important to note that MLN4924 is currently undergoing multiple Phase 1b trials to determine its safety and feasibility in combination with conventional chemotherapy for CRC treatment of CRC (Zhao et al. 2014).

Strategies for targeted cancer therapy in the clinic also include the development of IAP protein inhibitors. Ceramide and its analog LCL85 are potent sensitizers of Fas-mediated apoptosis and inhibit cancer progression by effectively targeting the protein degradation of cIAP1 and XIAP(Paschall et al. 2014). Tolinapant (ASTX660), a novel IAP antagonist, efficiently and rapidly downregulated cIAP1 expression in a CRC model. Additionally, FOLFOX was shown to promote tolinapant-induced apoptosis in human CRC and murine organ models, providing evidence for the clinical exploration of tolinapant in combination with FOLFOX for the treatment of cIAP1-expressing CRC with poor prognosis and high microsatellite stability (Crawford et al.^2021^).

Currently, there are other inhibitors of ubiquitination enzymes that have proven to be potential therapeutics for CRC. SMI#9 is a potent novel RAD6-selective small-molecule inhibitor that targets the RAD6 catalytic site to affect translesion synthesis (TLS) and enables CRC to overcome resistance to L-OHP and is required to overcome cisplatin-induced replication fork stalling (Sanders et al.^2017^). CC0651, an inhibitor that selectively inhibits the E2 enzyme Cdc34, has also been found to effectively inhibit p27Kip1 ubiquitination via E3 ligase SCF^Skp2^ (Ceccarelli et al. 2011). Further studies revealed that CC0651 and its analogs promoted p27 accumulation and inhibited the proliferation of CRC (Ceccarelli et al.^2011^). Another inhibitor that effectively inhibits APC-Cdh1 activation, in addition to inducing cell cycle arrest, is pro-TAME (Zeng et al. 2010).

The newest technology developed to connect cell surface E3 ubiquitin ligases to transmembrane proteins for degradation is protein hydrolysis-targeted antibodies (PROTABs). Antibodies targeting zinc finger and ring finger 3 (ZNRF3) protein hydrolysis allow for “on demand” degradation specific to CRC. Currently, one of the drawbacks of targeting E3 ubiquitin ligase in the clinic is the lack of specific targeting agents, leading to unexpected toxicity and side effects in clinical treatment. The experimental finding of targeting E3 ubiquitin ligase is of great benefit for cancer treatment. Several high-throughput screening assays are now available for the rapid filtration of small-molecule compounds from E3 ligases, providing a route to screen for clinically available E3 targeted drugs. An alternative approach is to directly target E3 ligase activity and antagonize the E3 ligase homolog of the target protein.

### Small molecules that specifically target ubiquitination processes in CRC

PS-341 is a proteasome inhibitor that blocks chemotherapy-induced NF-κB activation, resulting in IkappaB degradation via the ubiquitin–proteasome pathway. In addition, PS-341 was demonstrated to significantly increase the chemosensitivity of CRC cells to CPT-11,which provides new ideas for clinical treatment (Cusack et al. 2001).Herbal therapy targeting ubiquitination enzymes is also a highlight of drug-resistant CRC therapies. Aidi injections antagonize the activity of the ubiquitin–proteasome (UPS) system and inhibit the breakdown of cytotoxic proteins by binding to the ubiquitin proteasome (Stein et al.^2022^). Curcumin increased the interaction between calmodulin-1 (Cdh1) and Skp2, leading to ubiquitination and degradation of Skp2, overcoming resistance to 5-FU in CRC, and inducing apoptosis in CRC cells resistant to 5-FU, suggesting that curcumin is a potential chemo-candidate for the treatment of 5-FU resistant CRC (Gan et al. 2023).

## Discussion

In conclusion, ubiquitination enzymes, pivotal components in the ubiquitination process, have demonstrated a critical involvement in the development of chemotherapeutic resistance in CRC. The mechanism by which ubiquitination enzymes contribute to chemotherapeutic resistance primarily revolves around their impact on various signaling pathways, including the Wnt/β-catenin signaling pathway, EMT, and disruption of the cell cycle. It is worth noting that currently approved clinical drugs for CRC chemotherapy, namely 5-FU, CPT-11, L-OHP, and cetuximab, are utilized for this purpose. Emerging evidence shows that aberrant ubiquitination is involved in chemotherapeutic resistance in CRC. Moreover, several small-molecule inhibitors are currently under development, but none have demonstrated sufficient efficacy in clinical trials.

At present, our investigations of ubiquitination enzymes are fragmented, and the role of each member in chemotherapeutic resistance and its precise mechanism of regulation are not fully understood. The recognition of the role of ubiquitination enzymes in chemotherapeutic resistance has been growing alongside the increasing number of studies conducted on these enzymes in various cancers. A notable example is the promotion of resistance to cisplatin in non-small cell lung cancer through TRIM17-mediated ubiquitination and degradation of RBM38 (Zhong et al. 2023);RNF6 promotes cisplatin resistance by transcriptionally activating the expression of proliferating cell nuclear antigens and attenuating DNA damage in lung adenocarcinoma (Sun et al. 2022).In addition, UBE2T promotes temozolomide resistance in glioblastoma by modulating the Wnt/β-catenin signaling pathway (Wang et al. 2023). The results of these experiments may provide insights into the mechanisms of chemoresistance of these ubiquitination enzymes in CRC. Thus, further investigation of each ubiquitination enzyme member is warranted to elucidate its relevance to chemotherapeutic resistance in CRC.

Moreover, the additional exploration of chemotherapeutic resistance caused by ubiquitination enzymes could potentially contribute to the discovery of therapeutic targets, the development of innovative inhibitors, and the formulation of novel therapeutic approaches in clinical settings. In this context, risperidone acts as a pharmacological inhibitor of TRAF4, effectively suppressing the self-renewal of glioblastoma (GBM), eradicating tumor pathogenicity, and reversing resistance to temozolomide (Li et al. 2022). TAK-243, a newly developed and highly targeted inhibitor of the E1 enzyme UAE, demonstrated its ability to effectively counteract resistance to bortezomib and carfilzomib in cell line models. Furthermore, it exhibited notable efficacy against primary cells obtained from patients suffering from relapsed/refractory myeloma. Additionally, TAK-243 displayed significant synergistic effects when combined with several anti-myeloma agents, such as doxorubicin, melphalan, and pabilastat (Zhuang et al. 2019). Although it has not been demonstrated that the E3 ubiquitin ligase Hakai is associated with chemoresistance, recent studies have shown that Hakin-1, a Hakai inhibitor, can inhibit EMT in CC, which may provide new insights into the treatment of chemoresistance (Martinez-Iglesias et al. 2020). In addition, several ubiquitination-associated inhibitors have been found to ameliorate side effects associated with chemotherapy. The anti-MDM2 inhibitor DS-5272 ameliorates cisplatin-induced nephrotoxicity by inhibiting NF-κB signaling (Fujikura et al. 2019).
